# Boosting Circularly Polarized Luminescence of Organic Conjugated Systems *via* Twisted Intramolecular Charge Transfer

**DOI:** 10.34133/2020/3839160

**Published:** 2020-04-22

**Authors:** Junfeng Li, Chenxi Hou, Chao Huang, Shanqi Xu, Xuelei Peng, Qi Qi, Wen-Yong Lai, Wei Huang

**Affiliations:** ^1^Key Laboratory for Organic Electronics and Information Displays, Institute of Advanced Materials (IAM), Nanjing University of Posts and Telecommunications, 9 Wenyuan Road, Nanjing 210023, China; ^2^School of Chemistry and Chemical Engineering, Southeast University, Nanjing 211189, China; ^3^Shaanxi Institute of Flexible Electronics (SIFE), Northwestern Polytechnical University (NPU), 127 West Youyi Road, Xi'an, 710072 Shaanxi, China

## Abstract

Realizing a high luminescence dissymmetry factor (*g*_lum_) is a paramount yet challenging issue in the research field of circularly polarized luminescence (CPL). Here, we reported a novel set of organic conjugated systems with twisted intramolecular charge transfer (TICT) characteristics based on conjugated *o*-carborane-binaphthyl dyads composing of binaphthyl units as chiral electron donors and *o*-carborane units as achiral electron acceptors, demonstrating intense CPL with large *g*_lum_ values. Interestingly, single-crystalline *o*-1 exhibited a high-level brightness and a large *g*_lum_ factor as high as +0.13, whereas single-crystalline *o*-2 processed a relatively low brightness with a decreased *g*_lum_ value to -0.04. The significant diversity of CPL-active properties was triggered by the selective introduction of *o*-carborane units onto the binaphthyl units. Benefiting from the large magnetic dipole transition moments in TICT states, the CPL activity of TICT *o*-carborane-based materials exhibited amplified circular polarization. This study provides an efficient molecular engineering strategy for the rational design and development of highly efficient CPL-active materials.

## 1. Introduction

In recent years, organic *π*-conjugated functional materials [1–3], especially chiroptical materials featuring with circularly polarized luminescence (CPL), have attracted growing interest for their wide potential applications in three-dimensional (3D) optical displays [4], optical storage and processing systems [5], color-image projection [6], liquid crystal lasers [7], biological probes and signatures [8], security tags [9], light-emitting diodes [10–12], and, especially, backlighting liquid crystal displays [13]. Concerning CPL-active materials, one of the major targets is to achieve a large luminescence dissymmetry factor (*g*_lum_), which reflects the level of CPL properties. Generally, *g*_lum_ = 2(*I*_L_ − *I*_R_)/(*I*_L_ + *I*_R_), in which *I*_L_ and *I*_R_ denote the intensity of left and right circularly polarized light, respectively [14]. Theoretically, *g*_lum_ is simply approximated by 4|*m*|*μ*|cos*θ*/(|*m*|^2^ + |*μ*|^2^), in which *m* and *μ* denote the magnetic and electric transition dipole moments, respectively, and *θ* denotes the angle between *m* and *μ* [15]. High *g*_lum_ values could only arise from *m*-allowed and *μ*-forbidden transitions, while low values are generally induced by *m*-forbidden and *μ*-allowed transitions. However, owing to the essential large ∣*μ*∣ and negligible ∣*m*∣ in chiral organic materials, it is generally very challenging to achieve highly efficient CPL activity. The development of a novel organic system featuring with relatively large ∣*m*∣ and depressed |*μ*| would contribute to enhancing CPL with large *g*_lum_.

To amplify *g*_lum_ values, various organic material systems and approaches have been explored by constructing aggregation-induced CPL material systems [16], self-assembly supramolecular material systems [17], Förster resonance energy transfer systems [18, 19], or triplet-triplet annihilation upconversion CPL systems [20], etc. Nevertheless, the |*g*_lum_| values of the existing organic systems are still quite low and generally fall into the range of 10^−4^ to 10^−2^, which hampers largely the further investigation of organic materials for CPL applications [21]. On the other hand, it has been well demonstrated that charge-transfer (CT) organic materials consisting of *π*-electron-rich donors and *π*-electron-deficient acceptors are endowed with a forbidden electron dipole transition moment and a relatively large magnetic transition moment [22]. It is surmised that intense CPL with large *g*_lum_ could thus be achieved for the CT-active materials that are emissive with inherent chirality, which remains yet to be attempted by far.

**Figure 1:**
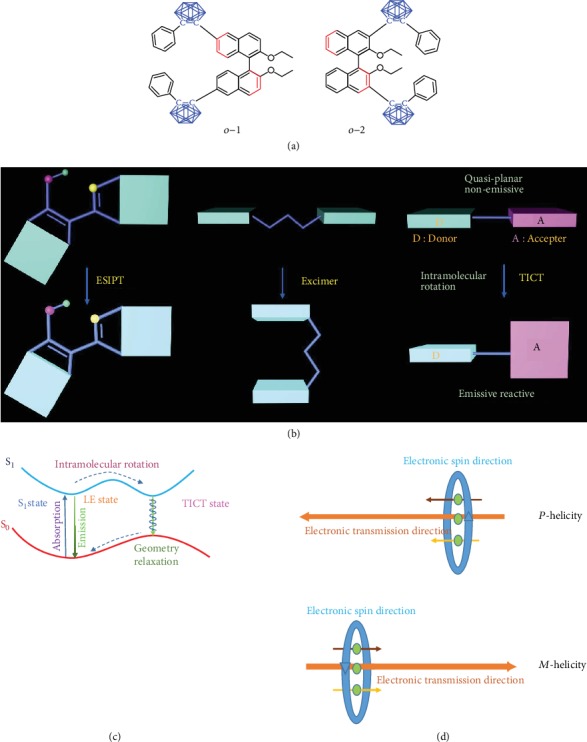
(a) Chemical structures of *o*-1 and *o*-2. (b, c) Schematic illustration of the twisted intramolecular charge transfer (TICT) mechanism, in comparison with those of the excited-state proton transfer (ESIPT) and excimer emission; “D” and “A” refer to electron-donating and electron-accepting units, respectively. (d) The relationship between electronic spin direction and molecular helical organization.

In this contribution, to verify the above hypothesis, a novel set of organic conjugated systems based on isomeric *o*-carborane-functionalized binaphthyl (BINOL) dyads with the same (*R*)-axial chirality have been designed, synthesized, and investigated, in which *o*-carborane units act as achiral electron acceptors and BINOL units as chiral electron donors. The chemical structures of the resulting *o*-carborane-binaphthyl dyads, 6,6′-carborane-substituted BINOL (*o*-1), and 3,3′-carborane-substituted BINOL (*o*-2), are depicted in Figure 1(a). The dihedral angles of BINOL are completely dependent on the substituents. The electron-deficient *o*-carborane units are prone to induce molecular charge transfer, leading to a charge-separated state [23, 24]. Twisted intramolecular charge transfer (TICT) emissions from *o*-1 to *o*-2 were observed, presumably originating from the rotational movement of *o*-carborane segments. The formation of the TICT state and the emission mechanism [25] are illustrated in Figures 1(b) and 1(c), in comparison with those of the excited-state proton transfer (ESIPT) [26] and excimer emission [27]. It is worthwhile to note that this represents the first example of CPL-active organic conjugated system with TICT characteristics. Moreover, single crystal *o*-1 exhibits intense CPL with a *g*_lum_ value of +0.13, which is attributed to the fine-tuning and precise control of dihedral angles of BINOL in the excited states. Substantially, the TICT process of *o*-carborane-based BINOL molecule is beneficial to regulate its dynamic conformations for enhancing magnetic transition dipole moments and forbidding electric transition dipole moments, thus boosting *g*_lum_ values.

## 2. Results and Discussion

The synthetic routes to *o*-1 and *o*-2 are depicted in Supplementary Materials. *o*-1 and *o*-2 were synthesized in an average yield about 53% by Diels-Alder cross-coupling reaction of decaborane with (*R*)-2,2′-diethoxy-6,6′-bis(phenylethynyl)-1,1′-binaphthyl and (*R*)-2,2′-diethoxy-3,3′-bis(phen- ylethynyl)-1,1′-binaphthyl using *N*, *N*-dimethylaniline as a Lewis base. The chemical structures of *o*-1 and *o*-2 were identified by ^1^H·NMR, ^13^C·NMR, and ^11^B·NMR spectroscopy as well as X-ray crystal data. Both are stable to H_2_O, air, and heat in solution and solid states. Thermogravimetric analysis (TGA) was employed to determine the thermal stability of *o*-1 and *o*-2. As shown in Figure S1, *o*-1 and *o*-2 exhibited good thermal stability with decomposition temperatures which started at approximately 417°C and 375°C, respectively, with a weight loss of 5% under a N_2_ atmosphere. Figures S2-3 demonstrated that *o*-1 and *o*-2 showed typical *π* − *π*^∗^ bands of the BINOL moieties in the range of 295 to 380 nm, devoid of effective conjugation between BINOL and the *o*-carborane moieties. Solvent-dependent TICT emissions were observed for *o*-1 and *o*-2, showing dual emissions in pure organic solvents [28]. With the increase of the solvent polarity, the emission bands of *o*-1 and *o*-2 exhibited noteworthy bathochromic shifts and their emission intensities decreased remarkably, indicating that the solvent polarity performed a paramount function in regulating the excited-state electronic conformation [29].

**Figure 2:**
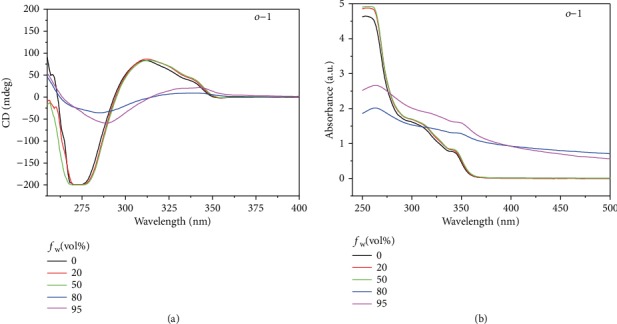
(a) CD and (b) UV-Vis spectra of *o*-1 in pure THF and H_2_O/THF solutions (*v*/*v*, 20 : 80, 50 : 50, 20 : 80, and 95 : 5). Solution concentration: 100 *μ*M.

To study the chiroptical properties of *o*-1 and *o*-2, the optical behaviors in aggregated states in the H_2_O-THF system were investigated. As shown in Figure S4, the solution-state emissions of *o*-1 and *o*-2 were hardly observed at the water fraction (*f*_w_) less than 90% with no aggregation that occurred. When *f*_w_ was up to 90%, the weak emission bands of *o*-1 and *o*-2 centered at 592 nm and 563 nm swiftly emerged, respectively. As *f*_w_ was 95%, the *λ*_em_ value of *o*-1 had an obvious blue shift by 31 nm with respect to *f*_w_ of 90%, while *o*-2 was only slightly blue shifted by 9 nm [28]. It was clearly demonstrated that the red-shifted spectra were attributed to the lowest TICT excited states [30]. On the other hand, the selective introduction of *o*-carborane units onto the BINOL skeletons also exerted influence on their aggregated patterns, resulting in different aggregation-induced emission- (AIE-) active properties. In order to explore the possible expression of chirality in aggregates and to obtain more chiral conformations of the assemblies in the ground states, circular dichroism (CD) spectra of *o*-1 and *o*-2 were performed in H_2_O/THF solutions. CD bands of *o*-1 and *o*-2 displayed an almost mirror image relationship. As depicted in Figure 2 and S5, *o*-1 and *o*-2 exhibited opposite signs in the first Cotton CD band at the position of the *π*-*π*^⁎^ band, suggesting that the bisignate Cotton effects came originally from the excitation couplings between obliquely oriented neighboring transition dipole moments [31]. In this regard, upon increasing the water fraction, the signal of CD has an obvious decrease and red shift, demonstrating a weak asymmetric nature of the self-assembly architecture of *o*-1. However, the CD signal of *o*-2 did not have an obvious change with the increasing water fraction in the H_2_O/THF solution. The major causes are that three-dimensional *o*-carborane substituents onto the BINOL skeletons lead to chirality inversion relative to BINOL. In this process, the conformation of *o*-2 underwent a portion of racemization. The positive CD couplet signified that *o*-1 had a predominantly *P*-chiral organization, while negative CD couplet of *o*-2 presented an *M*-chiral organization [32]. It proved that the chiral configuration of *o*-2 underwent a complete inversion relative to that of *o*-1 due to the selective introduction of three-dimensional *o*-carborane substituents onto the BINOL units. These results correlated well with the results from the UV spectra.

**Figure 3:**
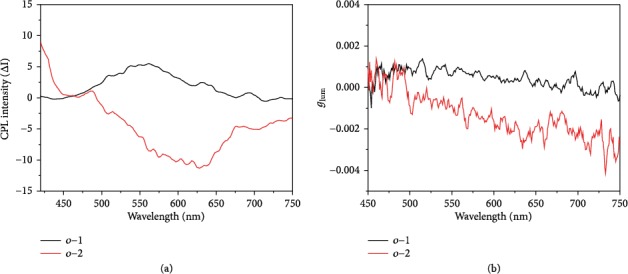
CPL and *g*_lum_ spectra of *o*-1 and *o*-2 in H_2_O/THF solution (*v*/*v*, 95 : 5). Solution concentration: 10 *μ*M.

To quantitatively access the level of CPL, the degree of CPL is evaluated using the dimensionless Kuhn's anisotropy factor (*g*_lum_) in photoexcited states. CPL signals of *o*-1 and *o*-2 were measured when the water fraction was 95% in the H_2_O/THF solution. The CPL signal is sensitive to external stimuli and also regarded as a remarkable indicator of excited molecular structures. As depicted in Figure 3, the *g*_lum_ value for *o*-1 is +9.13 × 10^−4^ at 561 nm and for *o*-2 is −1.81 × 10^−3^ at 627 nm, when *f*_w_ is up to 95%. Interestingly, *o*-1 and *o*-2 are almost mirror images, and the signs of the CPL spectra are reverse even though *o*-1 and *o*-2 have the same axially chiral BINOL unit. Indeed, the sign of the CPL spectra for *o*-1 is positive with a *P*-chiral organization, whereas that of *o*-2 is negative with a *M*-chiral organization. However, the detected absolute *g*_lum_ value decreased by one order of magnitude of *o*-1 compared to that of *o*-2, depending on the nature of the excited structures. One reason is that the irregular aggregated assembles are unfavorable for enhancing the *g*_lum_ values, as supported by scanning electron microscopy (SEM) images (Figure S7) [33]. It should be noted that it is readily available to record the CPL signals of *o*-1 and *o*-2 in the self-assemble states, and the detected CPL signals are mainly ascribed to the TICT emissions in the excited states. A dynamic excited molecular structure opens a way for regulating its magnetic and electric transition dipole moments, which is beneficial to boost the *g*_lum_ values. This can explain why the *g*_lum_ value for *o*-2 was larger than that of *o*-1 in the aggregated states.

**Figure 4:**
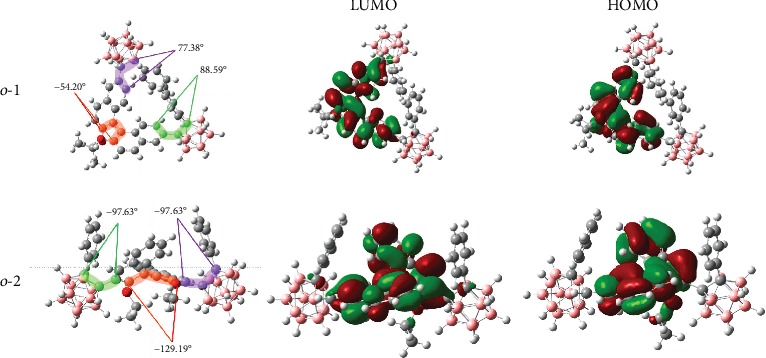
Optimized geometries and their frontier orbitals for S_1_ in *o*-1 and *o*-2 using the state-specific polarizable continuum model (PCM) at the TD-DFT CAM-B3LYP/6-31+G(d,p) level in the aggregated states.

To unravel the essence of the CPL sign reversal, the time-dependent density functional theory (TD-DFT) with the CAM-B3LYP functional was carried out. CD spectra are dominated by dihedral angles of the BINOL units through the investigation of chiroptical CD signs in the fluidic solution and solid states of BINOL luminophores with the same axial chirality. However, the specific relationship between CPL activity of TICT dyes and their excited conformations has been rarely reported even in aggregated states. While the optimized excited-state (*S*_1_) conformation of *o*-1 is characterized by a highly twisted BINOL core with a twisting angle (*θ*) of -54.20°, *o*-2 is found to be more planar, featuring a larger *θ* of -129.19° (Figure 4). It can be inferred that the conformation of *o*-2 underwent a reversion with respect to *o*-1 even though both compounds have the same axially chiral BINOL units [34]. The *g*_lum_ value of *o*-2 is larger than that of *o*-1 when *f*_w_ is 95%, revealing that the *g*_lum_ value is mainly determined by the aggregated electronic structure in the excited states. The highest occupied molecular orbital (HOMO) levels in both conformers are located almost on BINOL moieties, while the lowest unoccupied molecular orbital (LUMO) levels reside on BINOL moiety and the connected partial C-C bond in one *o*-carborane moiety, giving rise to a piece of direct evidence for the notable characteristic ICT behaviors. Hence, CPL caused by aggregated behaviors comes originally from the TICT states.

**Figure 5:**
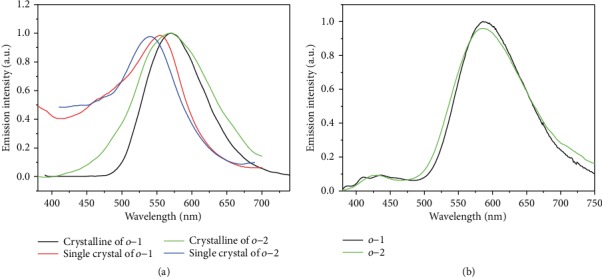
(a) Normalized emission spectra of *o*-1 and *o*-2 in the single-crystalline states and in the pristine-crystalline states at room temperature. (b) Normalized emission spectra of *o*-1 and *o*-2 in the crystalline states at 77 K.

The emission features of *o*-1 and *o*-2 crystallines are analyzed by photophysical spectroscopy and low-temperature emission spectra. As depicted in Figure 5(a), crystal powders of *o*-1 and *o*-2 exhibited a strong yellow emission with *λ*_max_ at 570 nm and 568 nm and a high *Φ*_EM_ of 85% and 78%, respectively, arising from the intrinsic TICT transitions from BINOL units to the C-C bond of *o*-carborane moieties. The emission band of single crystal *o*-1 exhibited a 16 nm hypochromatic shift from 570 nm to 554 nm, while *o*-2 showed a 29 nm hypochromatic shift from 568 nm to 539 nm. The results indicated that *o*-1 and *o*-2 possessed not only AIE but also crystallization-induced emission (CIE) properties due to the dynamic rotational movement of *o*-carborane units [35, 36]. To verify this assumption, the fluorescence spectra of *o*-1 and *o*-2 at 77 K were recorded in the crystalline states. Generally, the rotation of *o*-carborane is suppressed in frozen media resulting in LE emission. As shown in Figure 5(b), the new emission bands *o*-1 and *o*-2 were observed in the range of 380-480 nm with the vibrational peaks, which were attributed to the emission from the localized excited (LE) states. The other broadband was attributed to the TICT emissions at 547 nm [37], indicating that intramolecular rotations take place even in the crystalline states. As a result, solid-state emissions of *o*-1 and *o*-2 were obtained *via* TICT electronic transitions, originating from unidirectional movement of *o*-carborane moieties in the crystalline states.

**Figure 6:**
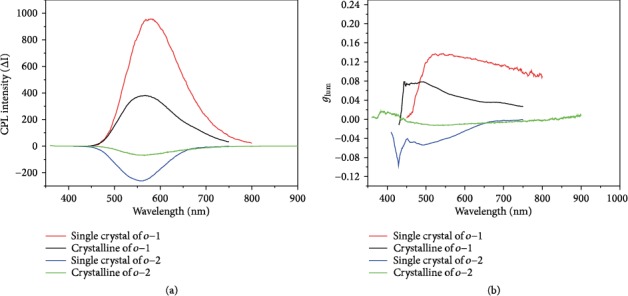
(a) CPL. (b) *g*_lum_ spectra of *o*-1 and *o*-2 in the single-crystalline states and in the pristine-crystalline states at room temperature.

As depicted in Figure S6, CD spectra of *o*-1 in the KBr-dispersed states displayed a bisignate Cotton effect with a positive wave at around 322 nm accompanied by a pronounced wavelength at around 360 nm and a negative wave at around 279 nm, indicating that chirality signal transferred from BINOL to *o*-carborane units. Meanwhile, a well-resolved bisignate CD signal of *o*-2 was observed with a positive wave at around 310 nm accompanied by a pronounced wavelength at around 247 nm and a negative wave at around 265 nm. These results also correlated well with the results of the UV spectra. The measurements of the solid-state CPL of *o*-1 and *o*-2 were carried out. As shown in Figure 6, *o*-1 and *o*-2 in crystal powders exhibit strong CPL emissions located at 563 nm and 562 nm, and their *g*_lum_ values are +0.05 and -0.01, respectively. Also, it is notable that, in single-crystalline states, the CPL emission bands of *o*-1 and *o*-2 centered at 578 nm and 556 nm, and their *g*_lum_ values are +0.13 and -0.04, respectively. The CPL signs agreed with those of the CD signals at the longest wavelengths, indicating that the ground and excited states exhibited similar conformations. Thus, the chirality signals of *o*-1 and *o*-2 inherently originated from *P*-chiral organization and *M*-chiral organization, respectively. The distinct difference of *g*_lum_ further confirms that *o*-1 may take a more twisted conformation in the excited states.

**Figure 7:**
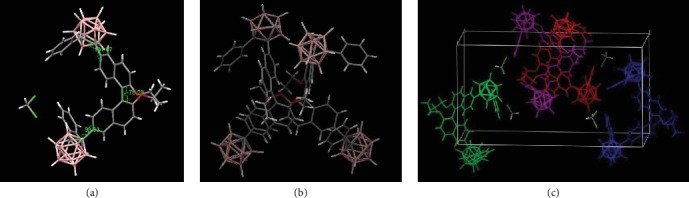
(a) ORTEP drawing, (b) intermolecular interactions, and (c) molecular packing of *o*-1.

**Figure 8:**
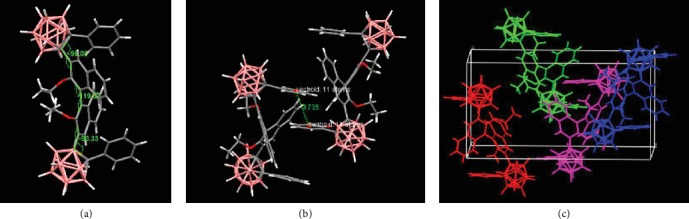
(a) ORTEP drawing, (b) intermolecular interactions, and (c) molecular packing of *o*-2.

To better understand the CPL properties of *o*-1 and *o*-2, crystals were obtained from the mixed solutions of methanol and dichloromethane. Their single-crystal structures were investigated in detail, and crystallographic data are given in Table S1-2. Both *o*-1 and *o*-2 adopted a three-dimensional twisted conformational structure in orthorhombic P2_1_2_1_2_1_. The data are summarized in Table S1-2. The dihedral angles between BINOL and the C-C bonds in the *o*-carborane units of *o*-1 are +106.67° and +93.33°, respectively. The BINOL angle is -75.29°. As shown in Figures 7 and 8, no distinct *π*-*π* stacking between BINOL units was found in the crystalline packing for *o*-1 and o-2, which facilitated the rotation of the *o*-carborane cluster to induce transformations among various conformation states. That is, the crystallization of *o*-1 takes a more twisted conformation than that of the aggregated states, which thus induces the TICT activity upon crystallization. The TICT states are responsible for CPL in the crystalline states, suggesting that the chirality signals transferred from BINOL to *o*-carborane units through space energy transfer [38]. As shown in Figure 8, the dihedral angles of BINOL in *o*-2 was -119.07°, meaning that intramolecular steric repulsion of *o*-2 was stronger than that of *o*-1. The CD intensities at the longest wavelengths are significantly affected by the dihedral angles. This explains why *o*-1 and *o*-2 show the same axial chirality but exhibit opposite signs in CD and CPL.

**Figure 9:**
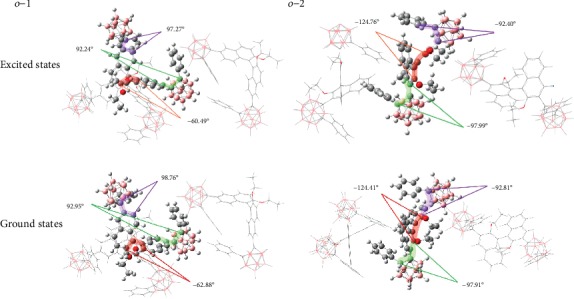
Optimized geometries for *S*_0_ and *S*_1_ of *o*-1 and *o*-2 using the state-specific PCM at the TD-DFT CAM-B3LYP/6-31+G(d,p) level in the crystalline states.

TD-DFT with the CAM-B3LYP functional of *o*-1 and *o*-2 in the crystalline states was also carried out to better understand the CPL properties. CD and CPL spectra are closely related to the dihedral angles of BINOL in ground states. As shown in Figure 9, the crystalline-phase calculations with the finite-size cluster model based on the single-crystal data predicted that the dihedral angles *θ* ((O)C-C-C-C(O)) of BINOL are obviously different (-62.88° for *o*-1, -124.41° for *o*-2), taking reversible signs in CD, whereas the dihedral angles *θ* ((O)C-C-C-C(O)) of BINOL are -60.49° and -124.76° for *o*-1 and *o*-2 in the excited states, respectively. Dihedral angles of BINOL units for *o*-1 and *o*-2 are dynamically regulated by the excitation, and the values of the angle change are 2.39° for *o*-1 and -0.35° for *o*-2. The results demonstrated that *o*-1 underwent a large dynamic conformational change upon *S*_0_⟶*S*_1_ excitation in the crystalline states. However, the conformational transitions upon *S*_0_⟶*S*_1_ excitation of *o*-2 were substantially suppressed in the crystalline phase. To put it simple, *o*-1 adopted a more twisted conformation than that of *o*-2, driven by the bulky substituents attached onto the open-type BINOL. There is no denying, though, that the detected larger *g*_lum_ value of *o*-1 in the crystalline state is ascribed to subtle variations in the excited-state conformations, resulting in an amplification of magnetic transition dipole moment, whereas the conformation of *o*-2 was almost not altering, leading to no alteration of the magnetic and electric transition dipole moments. Natural transition orbital (NTO) is employed to analyze a *π*-*π*^∗^ transition of the BINOL moiety and additional TICT contribution from the central BINOL donor to the peripheral *o*-carborane acceptor for *o*-1 and *o*-2, as shown in Figure S10 [39]. Hence, the nature of the CPL for *o*-1 and *o*-2 is the result of TICT states in the crystalline phases. Theoretically, *g*_lum_ is defined as 4|*m*|*μ*|cos*θ*/(|*m*|^2^ + |*μ*|^2^). Accordingly, high *g*_lum_ values could only be achieved for *m*-allowed and *μ*-forbidden transitions, while low values are generally expected for *m*-forbidden and *μ*-allowed transitions. To deduce the origin of these anomalous *g*_lum_ values, we performed TD-DFT analysis at the TD-TPSSTPSS/6-31G(d,p) level [40]. The magnetic and electric transition dipole moments from *S*_0_⟶*S*_1_ state for *o*-1 were 0.063 and 0.068, respectively, and for *o*-2 were 0.144 and 0.645, respectively. Meanwhile, the two transition dipole moments from *S*_1_⟶*S*_0_ state, *m* and *μ*, for *o*-1 (i.e., with an angle of 75.51°) were 0.800 and 1.002, respectively, and for *o*-2 (i.e., with an angle of 176.10°) were 0.153 and 1.887, respectively. These data showed that *o*-1 possessed an anomalously magnetic transition dipole moment in the *S*_1_⟶*S*_0_ state. The *g*_lum_ value is derived as a combination of |*m*| and |*μ*| in a relationship of *g*_lum_ by (|*m*|/|*μ*|cos*θ*). Thus, unlike conventional organic molecules with negligible magnetic transition dipole moment contributions, it stands to reason that the choice of 3,3′- and 6,6′-substituted *o*-carborane in *o*-1 and *o*-2 exerts a great influence on the molecular packing and electronic transmission, responsible for modulating the magnetic and electric transition dipole moments. The dynamic rotational movements of *o*-carborane moieties may lead to a small |*μ*| value and enhance the corresponding |*m*| value, arising an enhancement of the *g*_lum_ value [41]. This is why *o*-1 manifests a larger *g*_lum_ than that of *o*-2 in crystalline states [42]. Moreover, the choice of 3,3′- and 6,6′-substituted *o*-carborane in *o*-1 and *o*-2 can regulate their arrangement of dipoles in the crystalline and even resolve their electronic spin directions upon photoexcited states, which have a direct correction with their chirality organizations, as shown in Figure 1(d). This gives a direct explanation that *o*-1 and *o*-2 exhibit inverse signals.

## 3. Conclusions

To conclude, a set of CPL-active organic conjugated systems based on chiral *o*-carborane-binaphthyl dyads have been designed, synthesized, and systematically investigated. Both dyads, in the crystalline states, exhibited TICT emissions owing to the dynamic rotational movement of *o*-carborane units, which represents the first example of CPL-active organic material system with TICT characteristics. Moreover, single-crystal *o*-1 exhibits intense CPL with a boosting *g*_lum_ value approaching +0.13, the highest value ever recorded in organic conjugated systems by far. Controlling dihedral angles in BINOL units is responsible for regulating molecular conformations, and the electron-deficient *o*-carborane is beneficial to enhance the magnetic transition dipole moments and forbidding the electric transition dipole moments, thus promoting a larger *g*_lum_ value. This study provides an efficient molecular engineering strategy for the rational design and development of highly efficient CPL-active materials with boosting *g*_lum_ values.

## Appendix

The first example of circularly polarized luminescence- (CPL-) active organic conjugated system with twisted intramolecular charge transfer (TICT) characteristics is reported. Impressively, single-crystal *o*-1 exhibits intense CPL with a boosting *g*_lum_ value approaching +0.13, the highest value ever recorded in organic conjugated systems by far. The results provide an efficient molecular engineering strategy for the rational design and development of highly efficient CPL-active materials.

## Supplementary Materials

Supplementary Materials Supplementary materials such as the detailed description of materials used. Synthetic and analytical procedures. Crystal data for o-1 and o-2. UV-Vis, PL, and NMR spectra are presented. DFT calculation details and additional tables and figures related to this article are provided. X-ray crystallographic data in CIF for CCDC 1914133 for *o*-1 and 1914144 for *o*-2 can be obtained free of charge from the Cambridge Crystallographic Data Centre via http://ww.ccdc.cam.ac.uk/data_request/cif and from the authors.
