# Supplementary material for: Boosting Circularly Polarized Luminescence of Organic Conjugated Systems via Twisted Intramolecular Charge Transfer
Source: Research (Wash D C). 2020 Apr 22;2020:3839160. doi: 10.34133/2020/3839160 (PMC7193308; doi:10.34133/2020/3839160)
Supplement: Supplementary Materials — such as the detailed description of materials used. Synthetic and analytical procedures. Crystal data for o-1 and o-2. UV-Vis, PL, and NMR spectra are presented. DFT calculation details and additional tables and figures related to this article are provided. X-ray crystallographic data in CIF for CCDC 1914133 for o-1 and 1914144 for o-2 can be obtained free of charge from the Cambridge Crystallographic Data Centre via http://ww.ccdc.cam.ac.uk/data_request/cif and from the authors. [file 3839160.f1.pdf]

Supplementary Materials

Boosting Circularly Polarized Luminescence of Organic Conjugated Systems *via* Twisted Intramolecular Charge Transfer

Junfeng Li[1], Chenxi Hou[1], Chao Huang[1], Shanqi Xu[1], Xuelei Peng[1], Qi Qi[2], Wen-Yong Lai[1,3,*] and Wei Huang[1,3]

[1]Key Laboratory for Organic Electronics and Information Displays, Institute of Advanced Materials (IAM), Nanjing University of Posts Telecommunications, 9 Wenyuan Road, Nanjing 210023, China

[2]School of Chemistry and Chemical Engineering, Southeast University, Nanjing 211189, China

[3]Shaanxi Institute of Flexible Electronics (SIFE), Northwestern Polytechnical University (NPU), 127 West Youyi Road, Xi'an 710072, Shaanxi, China

Correspondence should be addressed to Wen-Yong Lai; iamwylai@njupt.edu.cn

Contents

1. Instruments and materials 3

2. Synthetic procedures and characterization 4

3. Thermo-gravimetric analysis 5

4. Photophysical properties 6

5. Scanning electron microscopy (SEM) 9

6. Chemical structure determination 9

8. NMR spectra 14

1. Instruments and materials

NMR spectra were recorded on a Bruker Ultra Shield Plus 400 MHz instruments with $CDCl_3$ as the solvent and tetramethylsilane (TMS) as the internal standard. UV-Vis spectra were obtained from a Perkin-Elmer Lambda 25 spectrometer. Fluorescent spectra were obtained with a 48000 DSCF spectrometer. Thermo-gravimetric analysis (TGA) was performed on Shimadzu DTG-60A equipment. Time-resolved fluorescence decays were obtained from HORIBA JOBIN YVON Tem Pro-01 lifetime fluorescence spectroscopy. Absolute fluorescence quantum yield was taken on Edinburgh Instruments' FLS980 fluorescence spectrophotometer attached with an integrating sphere. Circular dichroism (CD) spectra were recorded on Jasco J-810 spectropolarimeter. Circularly polarized luminescence (CPL) spectra in THF, THF/H_2O solution, a single crystal and polycrystal states were measured using a JASCO CPL-300 spectrofluoropolarimeter at room temperature. The instrument used a scattering angle of 0°C from the excitation of unpolarized, monochromated incident light with a bandwidth of 10 nm. Single-crystal X-ray diffraction data collection and structure determination of *o*-1 and *o*-2 were performed at 295 K with an Xcalibur Onyx Nova four-circle diffractometer with a CCD system utilizing graphite-monochromatic Cu Kα radiation (λ = 1.54184 Å). The empirical absorption correction was performed using the Crystal Clear program. The structure was solved using a direct method, and refined by full-matrix least-squares on F^2 employed in the program SHELXL-2016/6 program package. We employed the PLATON software/SQUEEZE subroutine to calculate the diffraction contribution of the solvent molecules and to generate a group of solvent-free diffraction intensities. The resulting new HKL file was used to further refine the structure. The crystallographic data and structure refinement parameters are summarized in Table S1-2. CCDC number 1914133 for *o*-1 and 1914144 for *o*-2 contains the supplementary crystallographic data for this paper. These data can be obtained free of charge from The Cambridge Crystallographic Data Centre.

THF was purified by distillation from sodium in presence of benzophenone. Other chemicals were analytical grades and used without further purification.

2. Synthetic procedures and characterization

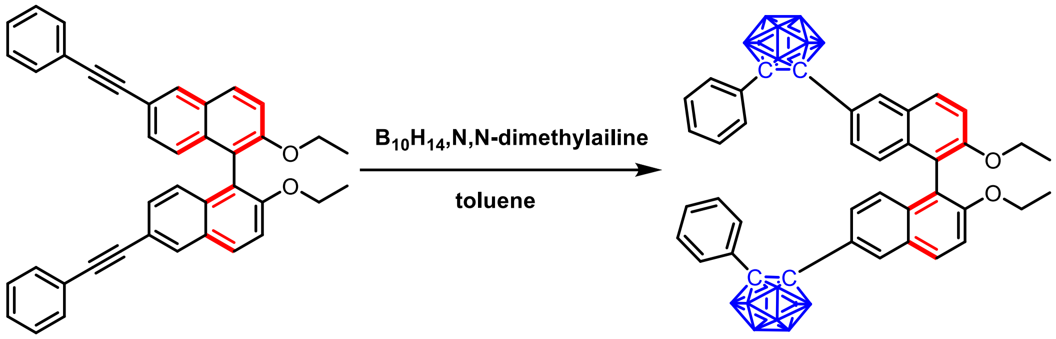

o-1

Synthesis of *o*-1: To a stirred toluene solution of decaborane ($B_{10}H_{14}$) (495.15 mg, 4.05 mmol) at room temperature was slowly added *N, N*-dimethylaniline (981.07 mg, 8.10 mmol), and then refluxed for 2 h. After being cooled to 40˚C, (*R*)-2,2'-diethoxy-6,6'-bis(phenylethynyl)-1,1'-binaphthyl (1.00 g, 1.84 mmol) was added in one portion and the mixture was stirred for 12 h. After being cooling to room temperature, the mixture was quenched by addition of methanol (20 mL). The organic phase was separated and the aqueous layer was extracted with CH_2Cl_2 (3 × 50 mL). The organic phases were combined, washed with brine, dried over anhydrous Na_2SO_4 and concentrated in *vacuo*. The residual was purified by column chromatography on silica gel (gradient of petroleum ether (bp. 60-90˚C)/EtOAc, 95/5 to 80/20, *v/v*) to afford the title product as a white powder (860.02 mg, 60%). The white single crystal was obtained from CH_2Cl_2/MeOH in 60% yield. ^1H NMR (400 MHz, $CDCl_3$): δ 7.92-7.91 (d, J = 4 Hz, 2H), 7.80 (s, 1H), 7.78 (s, 1H), 7.43-7.41 (m, 4H), 7.33 (s, 1H), 7.31 (s, 1H), 7.17-7.13 (m, 4H), 7.08-7.03 (m, 4H), 6.72 (s, 1H), 6.70 (s, 1H), 3.99-3.85 (m, 4H), 0.87-0.84 (t, 6H) ppm. ^{13}C NMR (101 MHz, $CDCl_3$): δ 155.46, 133.96, 131.43, 128.13, 126.99, 125.27, 118.79, 115.92, 85.83, 85.45, 67.98, 64.78, 25.62 ppm. ^{11}B NMR (128 MHz, $CDCl_3$): δ -2.37, -10.74 ppm.

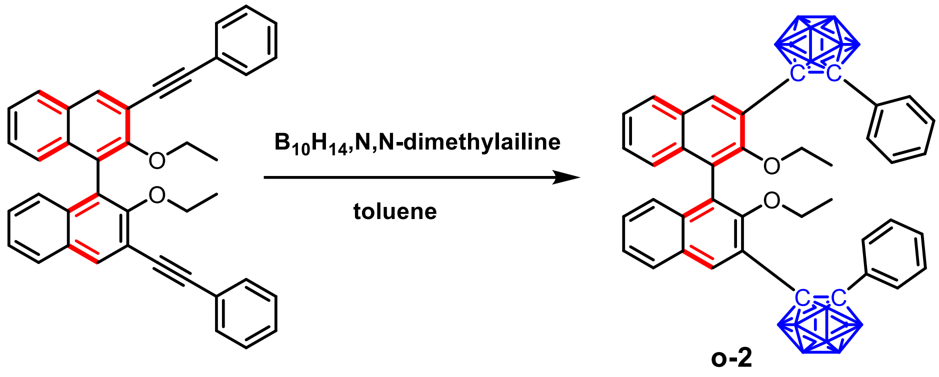

Synthesis of *o*-2: *o*-2 was prepared from (*R*)-2,2'-diethoxy-6,6'-bis(phenylethynyl)-1,1'-binaphthyl (1.00 g, 1.84 mmol) according to the procedure of that described for the preparation of *o*-1. Yield: 45% (645.01 mg, pure white solid). ^{1}H NMR (400 MHz, $CDCl_3$): δ 8.34 (s, 2H), 7.77-7.75 (d, J = 8 Hz, 2 H), 7.64-7.62 (d, J = 8 Hz, 4H), 7.37-7.33 (t, J = 8H, 2H), 7.27-7.18 (m, 8H), 6.66-6.64 (d, J = 8H, 2H), 3.20-3.13 (m, 2H), 3.03-2.96 (m, 2H), 0.47-0.43 (t, J = 8H, 6H) ppm. ^{13}C NMR (101 MHz, $CDCl_3$): δ 154.05, 137.56, 134.16, 131.37, 130.24, 128.85, 128.73, 128.34, 125.48, 125.10, 124.89, 123.71, 86.97, 84.65, 68.76, 14.65 ppm. ^{11}B NMR (128 MHz, $CDCl_3$): δ -1.40, -3.10, -10.26 ppm.

3. Thermo-gravimetric analysis

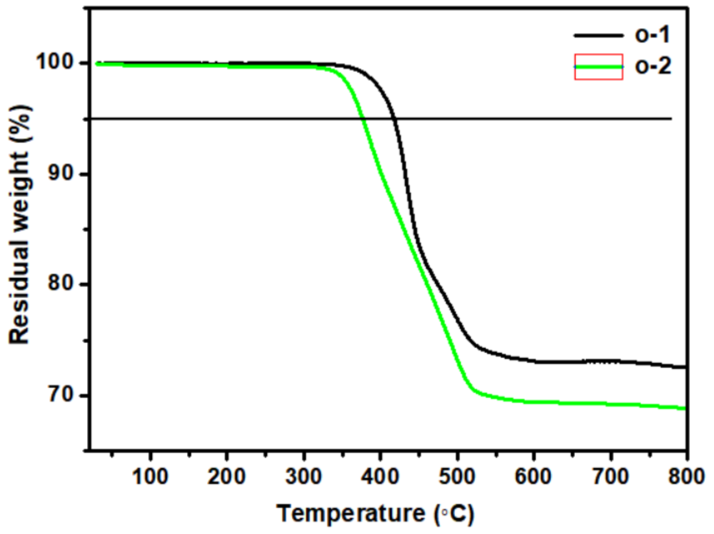

Figure S1. TGA curves of *o*-1 and *o*-2.

4. Photophysical properties

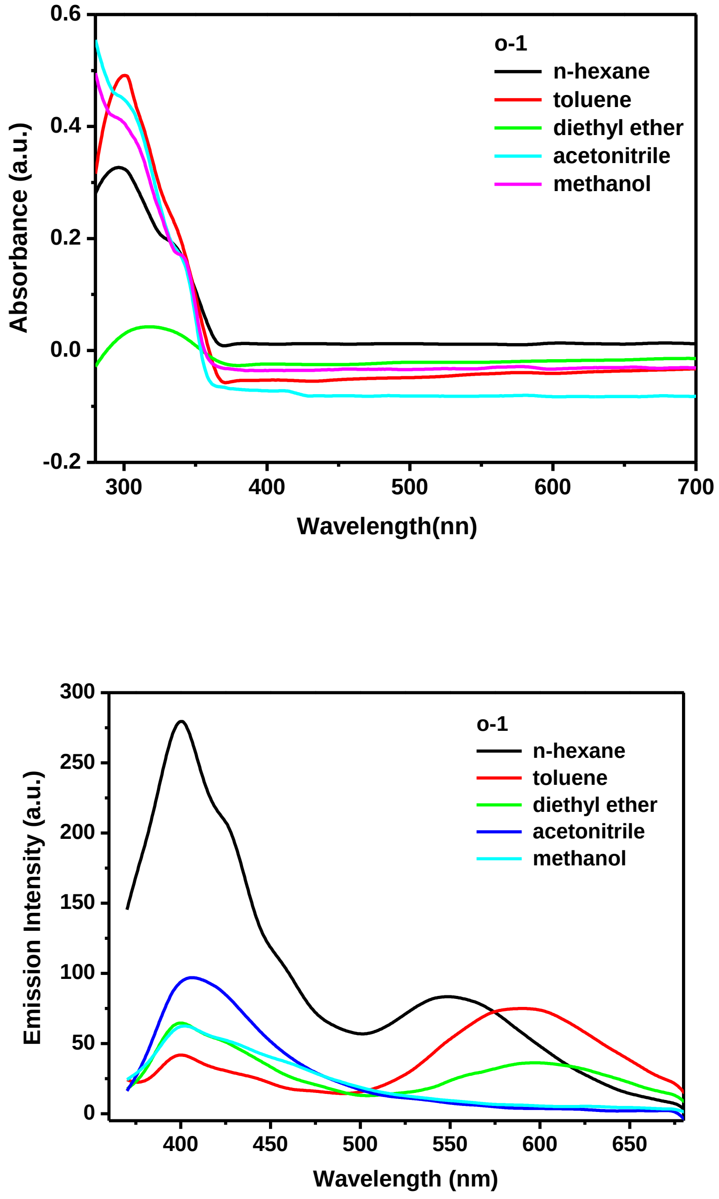

Figure S2. UV-Vis absorption and emission spectra of *o*-1 in various solvents. Solution concentration: 10 μM.

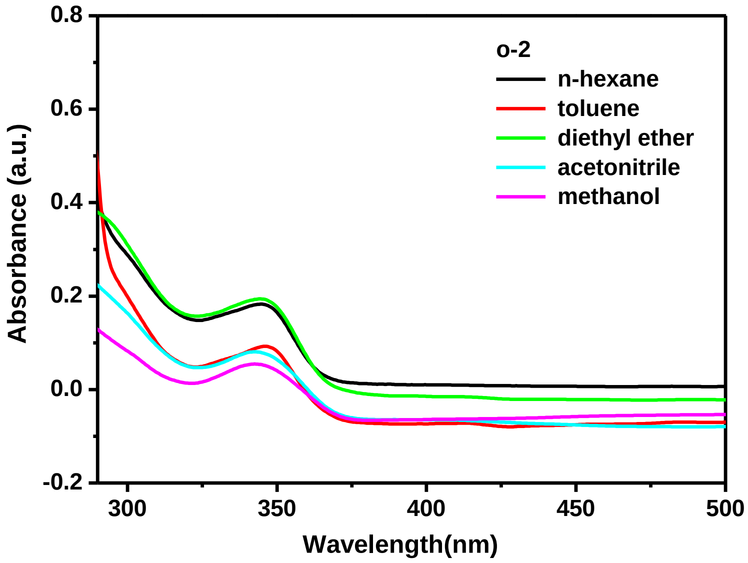

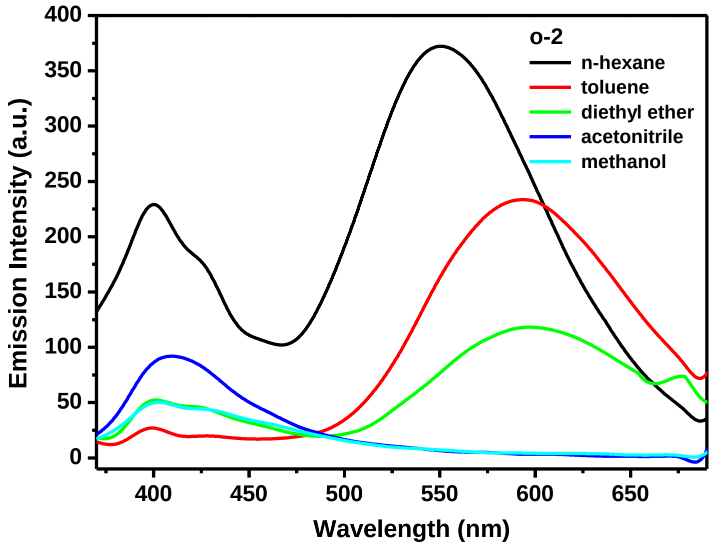

Figure S3. UV-vis absorption and emission spectra of *o*-2 in various solvents. Solution concentration: 10 μM.

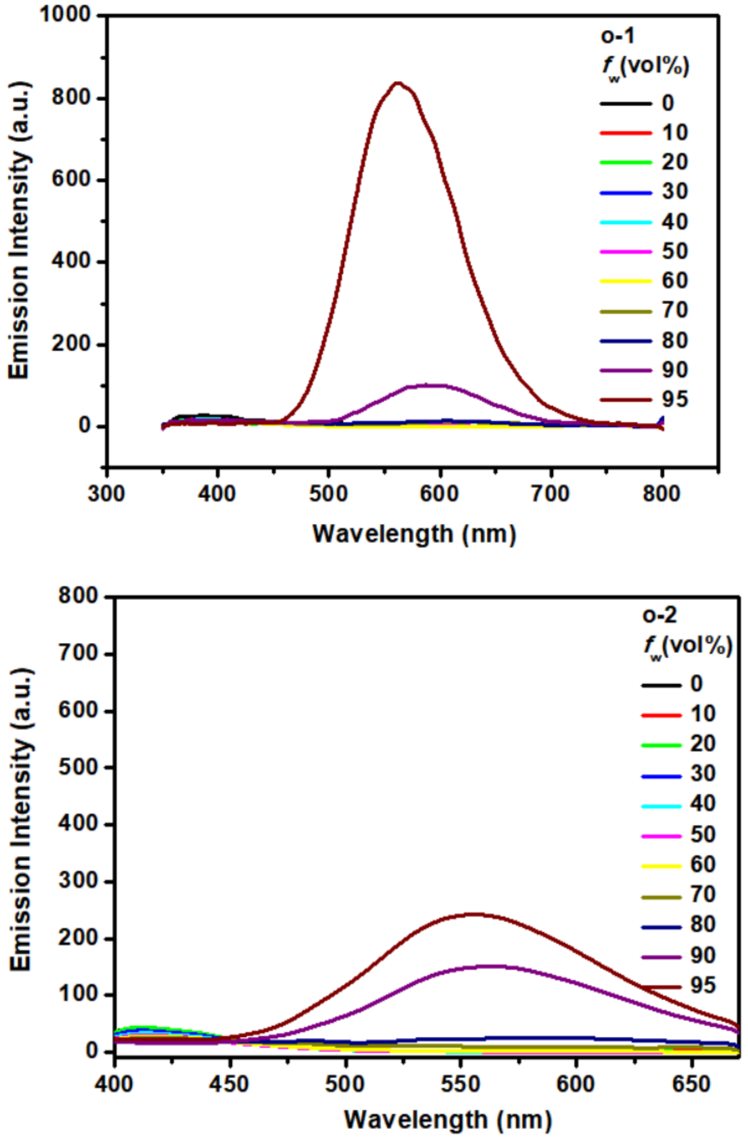

Figure S4. Emission spectra of *o*-1 and *o*-2 in THF, upon increasing the concentration water from 0% to 95% (λ_{ex} = 330 nm). Solution concentration: 10 μM.

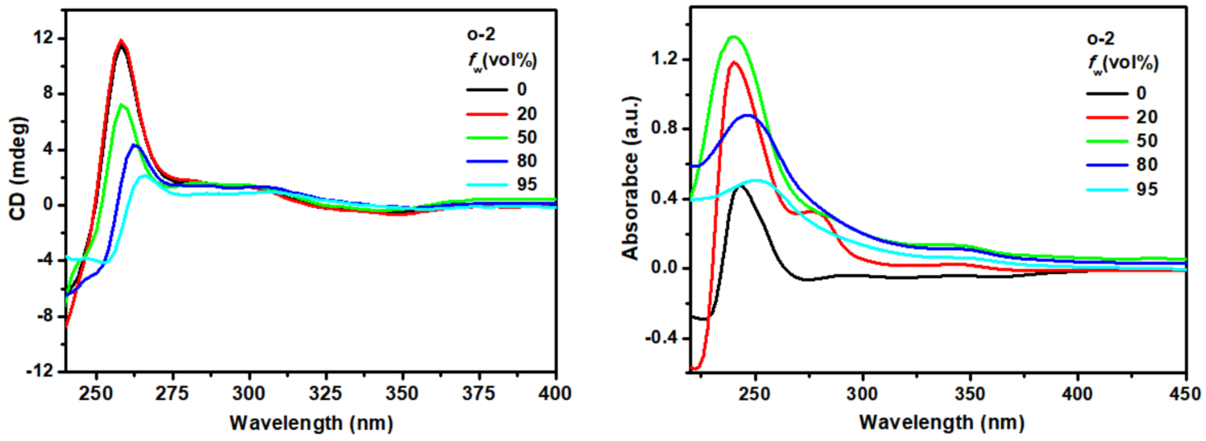

Figure S5. CD and UV-vis spectra of *o*-2 in pure THF and H_2O/THF solutions (*v/v*, 20:80, 50:50,

20:80, and 95:5). Solution concentration: 10 μM.

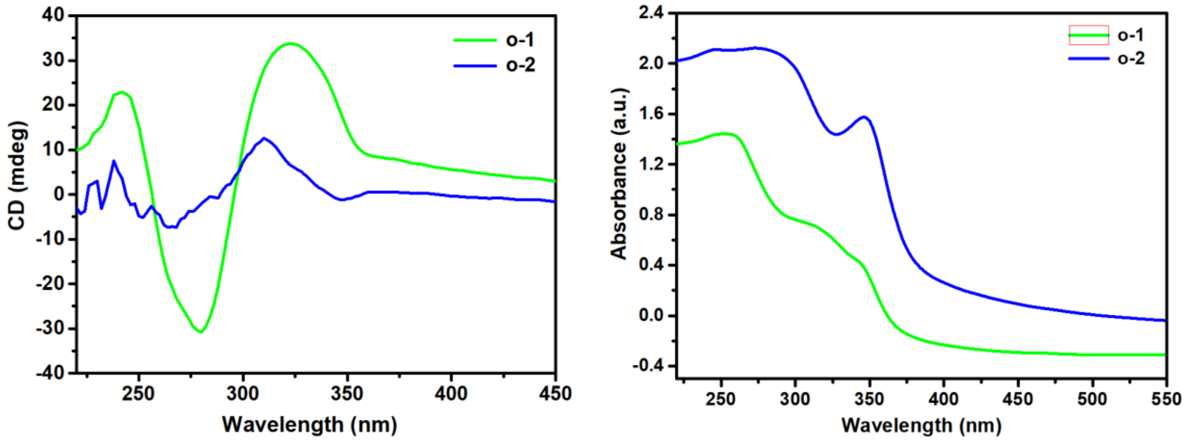

Figure S6. CD and UV-vis spectra of *o*-1 and *o*-2 dispersed in the KBr pellet.

5. Scanning electron microscopy (SEM)

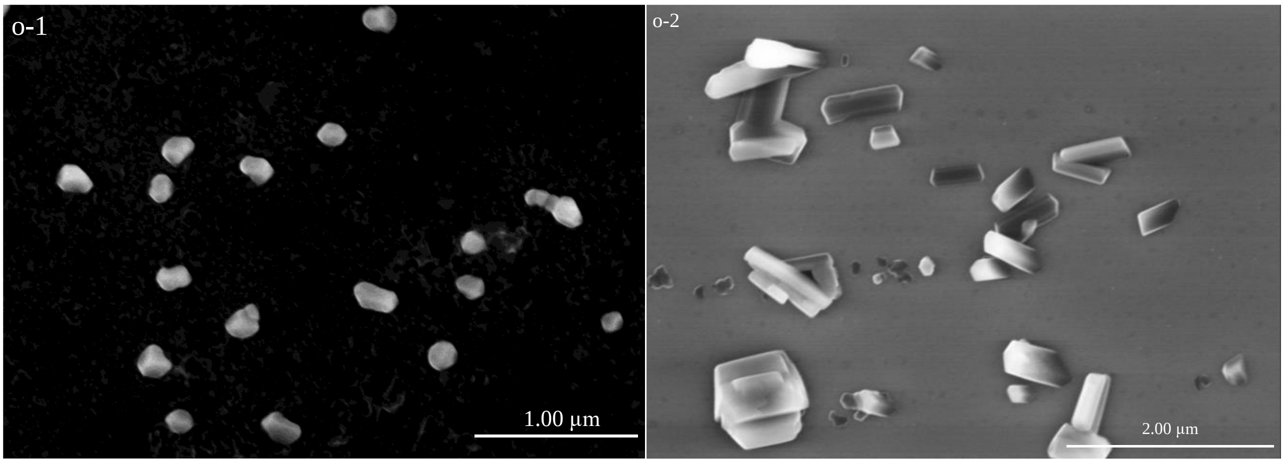

Figure S7. SEM images of *o*-1 and *o*-2 in H_2O/THF solution (v:v = 95:5) solution. Solution concentration: 10 μM.

6. Chemical structure determination

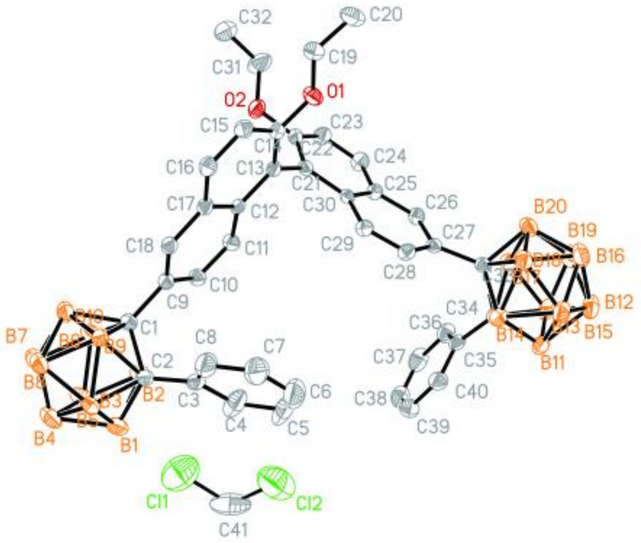

Figure S8. ORTEP drawing (30% probability for thermal ellipsoids) of *o*-1. The hydrogen atoms are omitted for clarity.

Table S1. Crystal Data and Structure Refinement for *o*-1.

	o-1
Empirical formula	$C_{41}H_{52}B_{20}Cl_2O_2$
Formula weight	863.93
Temperature (K)	295(2)
Wavelength	0.71073 Å
Crystal system, space group	Orthorhombic, $P2_12_12_1$
Unit cell dimensions	a = 10.5845(12) Å α = 90.00 °
	b = 15.7384(18) Å β = 90.00 °

	c = 29.027(3) (4) Å γ = 90.00 °
Volume	4835.4(9) Å^3
Z, Calculated density	4, 1.187 g/cm^3
μ	0.170 mm^{-1}
F(000)	1792
Crystal size	0.20 × 0.20 × 0.20 mm
θ range for data collection	1.40 to 28.25 °
Limiting indices	$-14 \le h \le 13$, $-20 \le k \le 20$,
	$-38 \le l \le 28$
Reflections collected / unique	43903 / 11941 [R_{int} = 0.0688]
Max. and min. transmission	0.967 and 0.967
Completeness to theta = 25.00	99.7%
Refinement method	Full-matrix least-squares on F^2
Data / restraints / parameters	11895 / 8 / 652
Absolute structure parameter	0.05(4)
Goodness-of-fit on F^2	1.020
Final R indices [I>2σ(I)]	R_1 = 0.0716, wR_2 = 0.1749
R indices (all data)	R_1 = 0.1338, wR_2 = 0.2068
Largest diff. peak and hole	0.338 and -0.292 e.Å^{-3}

$^aR_1 = \Sigma||F_o|-|F_c||$ (based on reflections with $F_o^2 > 2\sigma F^2$), $^bwR_2 = [\Sigma[w(F_o^2-F_c^2)^2]/\Sigma[w(F_o^2)^2]]^{1/2}$; $w = 1/[\sigma^2(F_o^2)+(0.095P)^2]$; $P = \max(F_o^2, 0)+2F_c^2]/3$(also with $F_o^2>2\sigma F^2$)

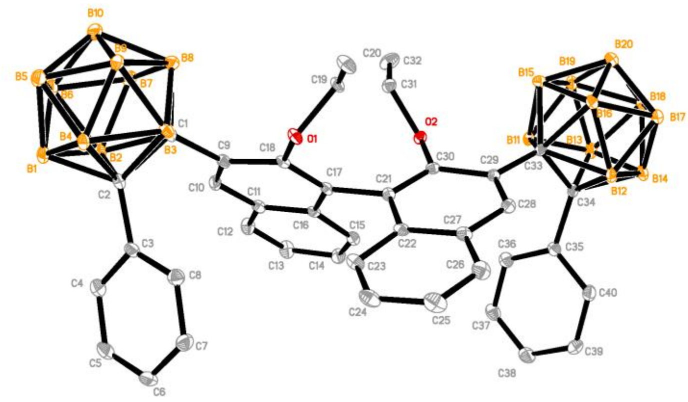

Figure S9. ORTEP drawing (30% probability for thermal ellipsoids) of *o*-2. The hydrogen atoms are omitted for clarity.

Table S2. Crystal Data and Structure Refinement for *o*-2.

	o-2
Empirical formula	$C_{40}H_{50}B_{20}O_2$
Formula weight	779.00
Temperature (K)	100(2)
Wavelength	0.71073 Å
Crystal system, space group	Orthorhombic, $P2_12_12_1$
Unit cell dimensions	a = 11.9834(12) Å α = 90.00 °
	b = 15.8885(15) Å β = 90.00 °
	c = 23.060(2) Å γ = 90.00 °
Volume	4390.7(7) $Å^3$
Z, Calculated density	4, 1.179 g/cm^3
μ	0.063 mm^{-1}
F(000)	1624
Crystal size	0.20 × 0.20 × 0.20 mm
θ range for data collection	1.556 to 28.334 °
Limiting indices	-15<=h<=16, -21<=k<=21, -23<=l<=30
Reflections collected / unique	40376 / 10966 [R(int) = 0.0674]
Max. and min. transmission	0.967 and 0.967
Completeness to theta = 25.25	100%
Refinement method	Full-matrix least-squares on F^2

Data / restraints / parameters	10913 / 0 / 562
Absolute structure parameter	0.0(6)
Goodness-of-fit on F^2	1.001
Final R indices [I>2σ(I)]	R_1 = 0.0536 , wR_2 = 0.1120
R indices (all data)	R_1 = 0.0769 , wR_2 = 0.1202
Largest diff. peak and hole	0.253 and -0.261 e.Å^{-3}

$^{a}R_1 = \Sigma||F_o|-|F_c||$ (based on reflections with $F_o^2>2\sigma F^2$), $^{b}wR_2 = [\Sigma[w(F_o^2-F_c^2)^2]/\Sigma[w(F_o^2)^2]]^{1/2}$;

$w = 1/[\sigma^2(F_o^2)+(0.095P)^2]$; $P = \max(F_o^2, 0)+2F_c^2]/3$(also with $F_o^2>2\sigma F^2$

7. Theoretical calculation

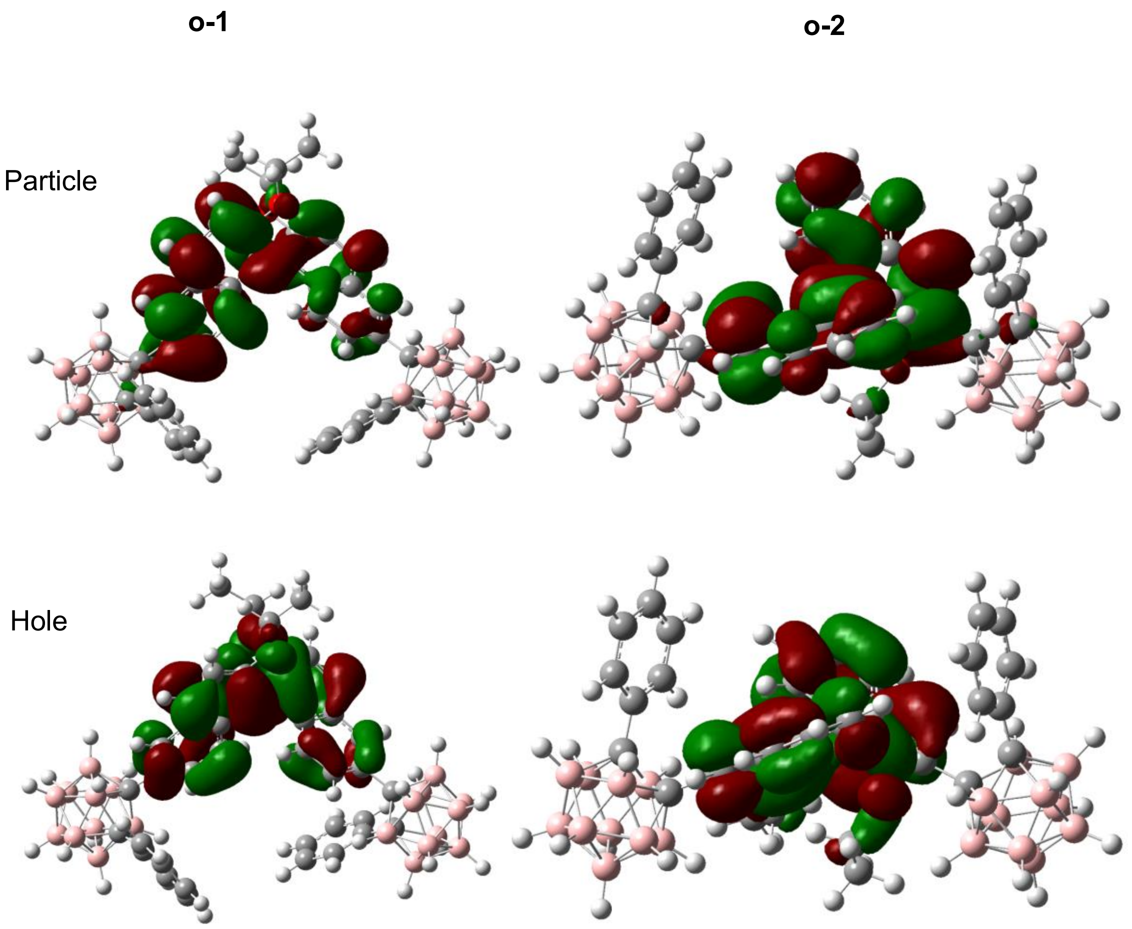

Figure S10. NTOs for the optimized S1 conformations of *o*-1 and *o*-2 in the crystalline phases.

8. NMR spectra

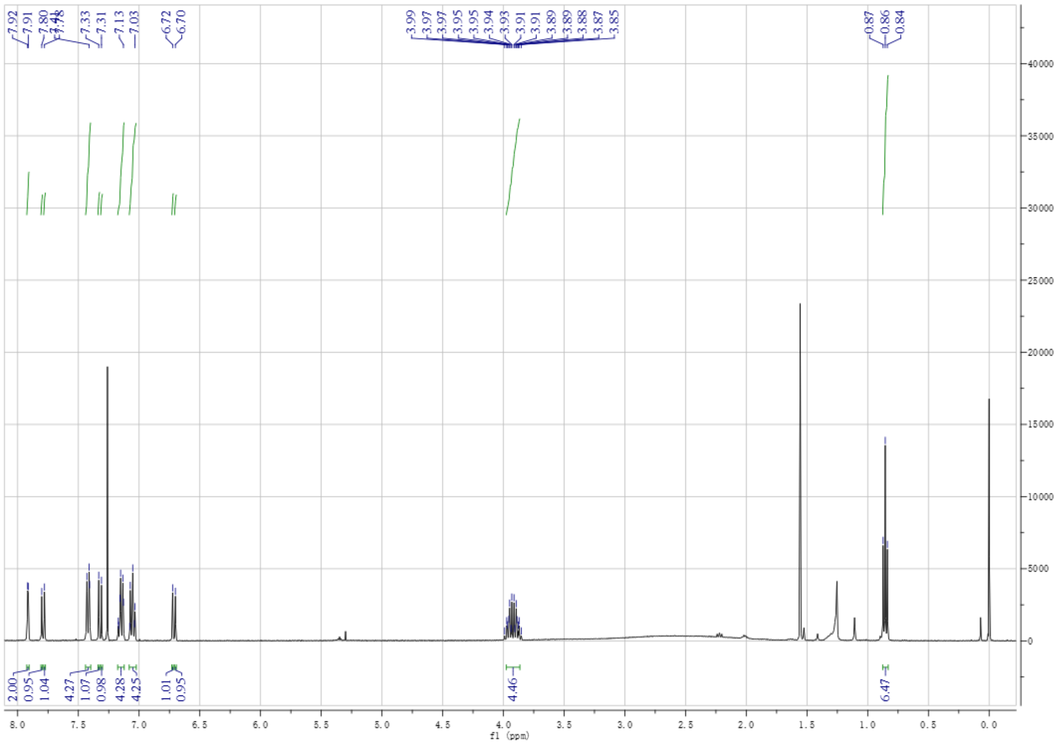

Figure S11. ^{1}H NMR spectra of *o*-1 in $CDCl_3$.

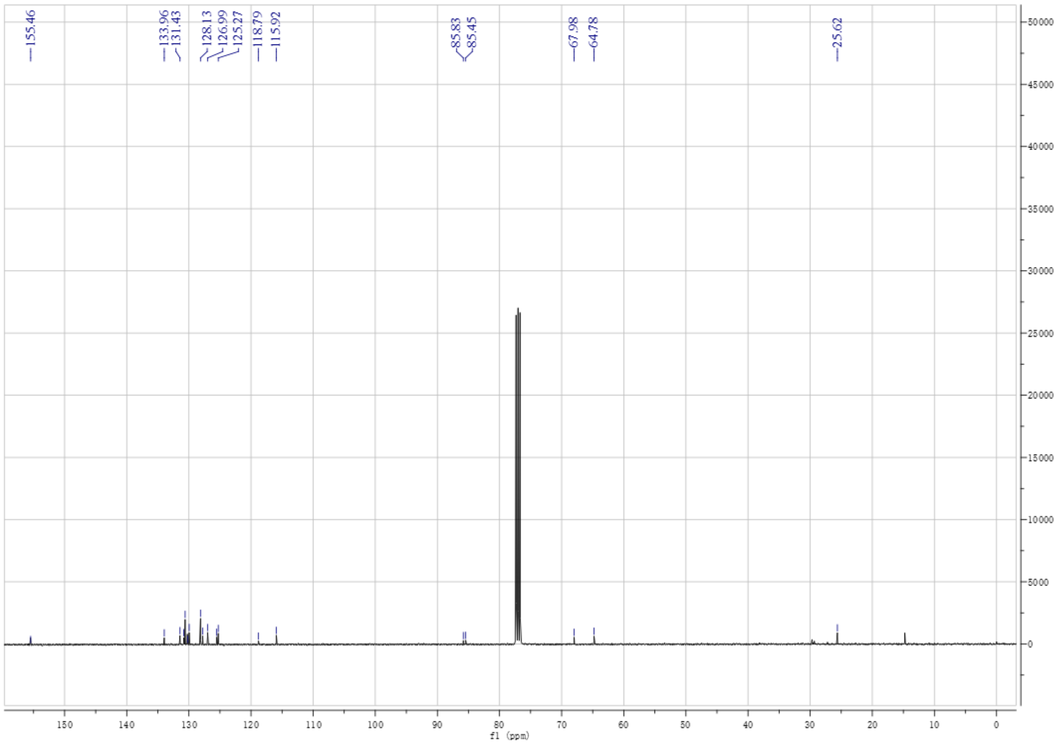

Figure S12. ^{13}C NMR spectra of *o*-1 in $CDCl_3$.

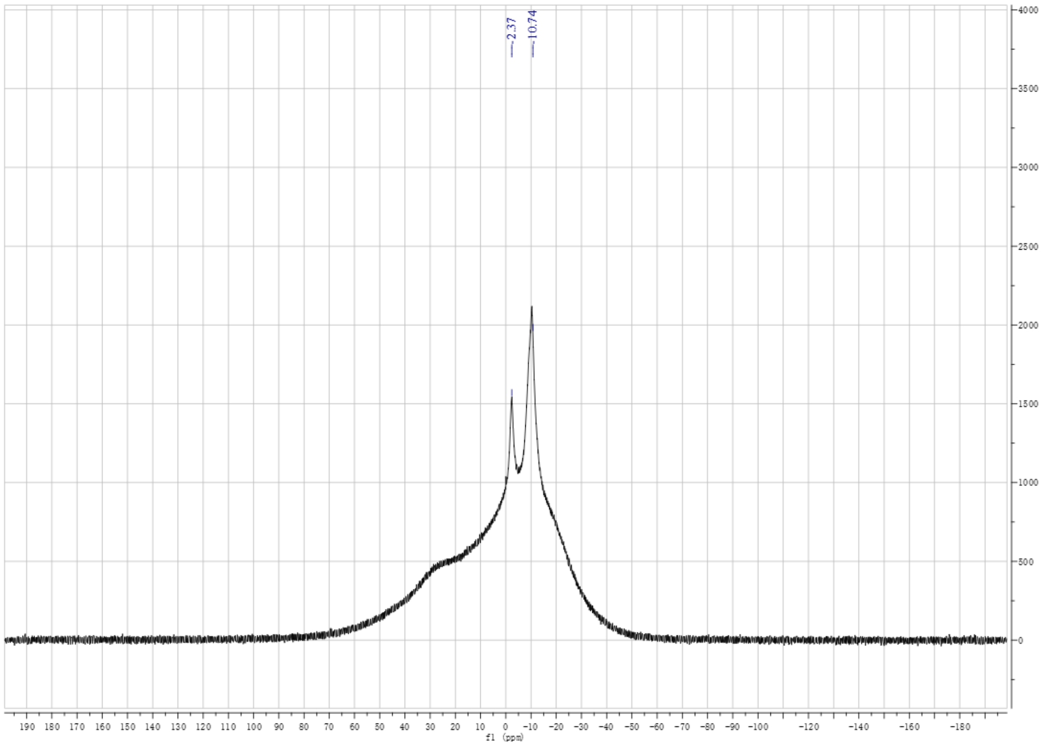

Figure S13. ^{11}B NMR spectra of *o*-1 in $CDCl_3$.

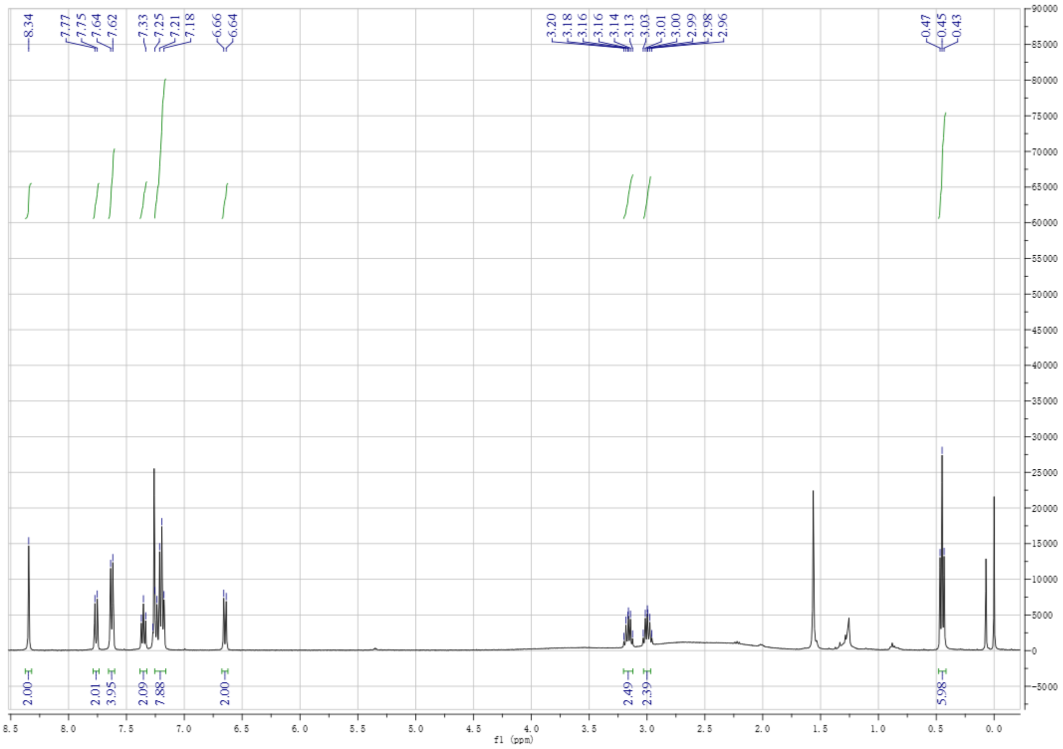

Figure S14. ^{1}H NMR spectra of *o*-2 in $CDCl_3$.

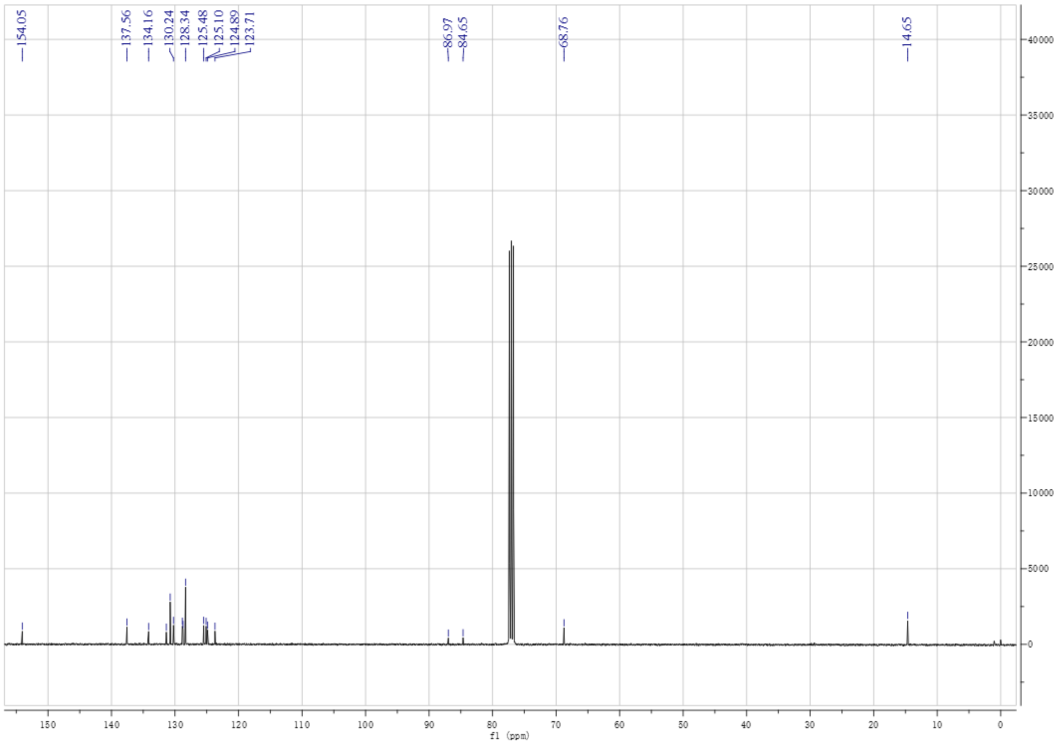

Figure S15. ^{13}C NMR spectra of *o*-2 in $CDCl_3$.

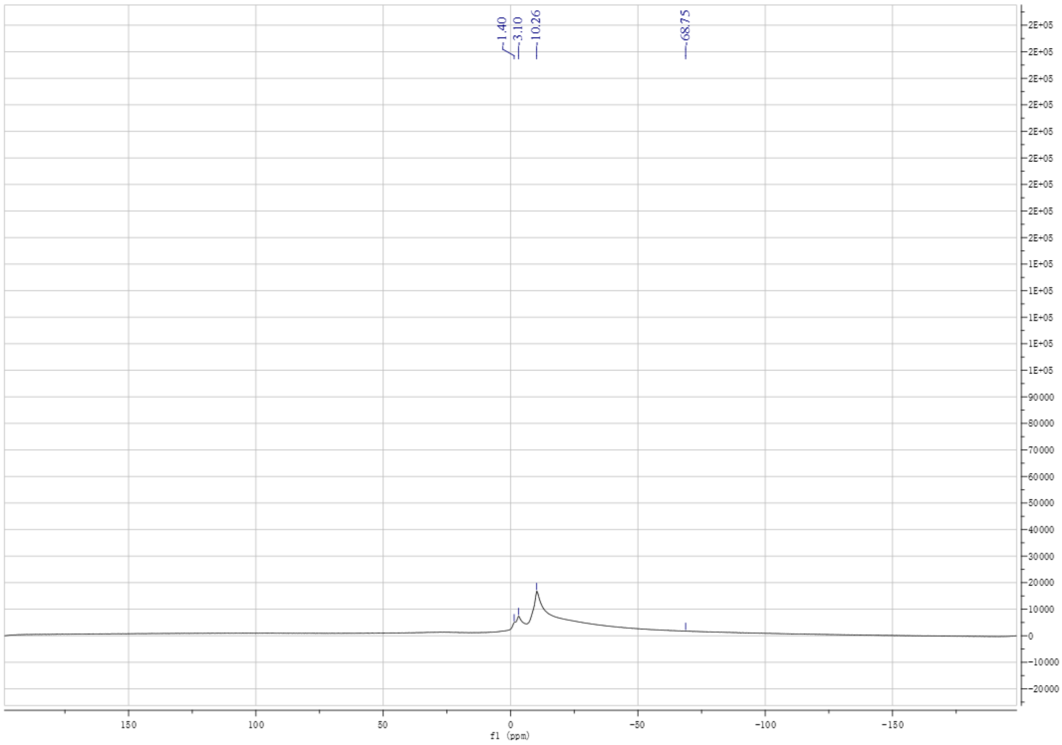

Figure S16. ^{11}B NMR spectra of *o*-2 in $CDCl_3$.
